# Ca^2+^-permeable AMPA receptors in mouse olfactory bulb astrocytes

**DOI:** 10.1038/srep44817

**Published:** 2017-03-21

**Authors:** Damian Droste, Gerald Seifert, Laura Seddar, Oliver Jädtke, Christian Steinhäuser, Christian Lohr

**Affiliations:** 1 Division of Neurophysiology, University of Hamburg, 20146 Hamburg, Germany; 2 Institute of Cellular Neurosciences, Medical Faculty, University of Bonn, 53105 Bonn, Germany

## Abstract

Ca^2+^ signaling in astrocytes is considered to be mainly mediated by metabotropic receptors linked to intracellular Ca^2+^ release. However, recent studies demonstrate a significant contribution of Ca^2+^ influx to spontaneous and evoked Ca^2+^ signaling in astrocytes, suggesting that Ca^2+^ influx might account for astrocytic Ca^2+^ signaling to a greater extent than previously thought. Here, we investigated AMPA-evoked Ca^2+^ influx into olfactory bulb astrocytes in mouse brain slices using Fluo-4 and GCaMP6s, respectively. Bath application of AMPA evoked Ca^2+^ transients in periglomerular astrocytes that persisted after neuronal transmitter release was inhibited by tetrodotoxin and bafilomycin A1. Withdrawal of external Ca^2+^ suppressed AMPA-evoked Ca^2+^ transients, whereas depletion of Ca^2+^ stores had no effect. Both Ca^2+^ transients and inward currents induced by AMPA receptor activation were partly reduced by Naspm, a blocker of Ca^2+^-permeable AMPA receptors lacking the GluA2 subunit. Antibody staining revealed a strong expression of GluA1 and GluA4 and a weak expression of GluA2 in periglomerular astrocytes. Our results indicate that Naspm-sensitive, Ca^2+^-permeable AMPA receptors contribute to Ca^2+^ signaling in periglomerular astrocytes in the olfactory bulb.

It has become increasingly evident during the past decade that astrocytes are far more than supportive cells in the brain, but rather take active part in information processing such as synaptic transmission and synaptic plasticity^1^,^2^. Most of the functions attributed to astrocytes are governed by cytosolic Ca^2+^ signaling^3^,^4^. Astrocytes are equipped with a plethora of receptors for neurotransmitters, neuropeptides and growth factors, most of which are linked to Ca^2+^ release from internal stores^5^. Hence, it is generally accepted that internal Ca^2+^ release is the main player in glial cell physiology. Recent studies, however, challenge this notion and demonstrate pivotal roles of Ca^2+^ influx in spontaneous Ca^2+^ signaling and in astrocyte function such as neurovascular coupling^6^,^7^. Astrocytes express Ca^2+^-permeable ion channels such as ionotropic neurotransmitter receptors, transient receptor potential channels and store-operated Ca^2+^ channels that mediate Ca^2+^ influx from the extracellular space^3^,^5^,^6^,^7^. For example, Bergmann glial cells, specialized astrocytes in the cerebellum, possess ionotropic glutamate receptors of the AMPA type that consist of GluA1 and GluA4 subunits, but lack the GluA2 subunit and hence exhibit high Ca^2+^ permeability^8^,^9^. Artificial insertion of the GluA2 subunit renders AMPA receptors in Bergmann glial cells Ca^2+^-impermeable and results in retraction of glial processes from synapses of Purkinje cells and abnormal synaptic currents^10^. This phenotype was mimicked in glia-specific GluA1/GluA4 double knock-out mice, which additionally showed impairment in fine motor coordination, emphasizing a pivotal role of Ca^2+^-permeable AMPA receptors in neuron-glia interactions^11^. In other brain regions such as the thalamus, astrocytes possess AMPA receptors of different subunit composition including variable contribution of the GluA2 subunit to the channel assembly and hence display intermediate Ca^2+^ permeability^12^. In the hippocampus, NG2 glial cells express AMPA receptors, while astrocytes lack these receptors^13^,^14^,^15^,^16^.

In the olfactory bulb, activation of neurotransmitter receptors in astrocytes has been shown to result in Ca^2+^ transients^17^. The olfactory bulb is the first relay station of odor information processing and is targeted by axons of sensory neurons in the olfactory epithelium, the olfactory receptor neurons. Olfactory receptor neurons release glutamate and ATP in neuropilar regions called glomeruli^18^,^19^, where they stimulate Ca^2+^ signaling in periglomerular astrocytes by mGluR_5_ and P2Y_1_ receptors^20^,^21^. In addition, ATP is degraded to adenosine, acting on astrocytic A_2A_ receptors^21^. Ca^2+^ increases in olfactory bulb astrocytes have been reported to release ATP from astrocytes and to trigger vasoresponses in blood vessels contacted by astrocytic end feet^20^,^22^,^23^,^24^. All Ca^2+^ responses to neurotransmitters measured in olfactory bulb astrocytes were mediated by Ca^2+^ release from internal stores, while Ca^2+^ influx from the extracellular space has not been demonstrated so far^17^. In the present study, we were interested whether olfactory bulb astrocytes express Ca^2+^-permeable AMPA receptors and whether these receptors are activated by glutamate released from olfactory receptor neurons. We studied Ca^2+^ responses and membrane currents in olfactory bulb astrocytes to application of AMPA and kainate, using Ca^2+^ imaging in brain slices and whole-cell patch-clamp recordings in acutely isolated astrocytes. Kainate-evoked membrane currents as well as Ca^2+^ transients induced by AMPA and electrical stimulation of olfactory receptor axons were partly reduced by Naspm (N-[3-[[4-[(3-aminopropyl) amino] butyl] amino] propyl]-1-naphthaleneacetamide trihydrochloride), an AMPA receptor blocker selective for GluA2-lacking, Ca^2+^-permeable AMPA receptors. Immunohistological staining revealed expression of GluA1, GluA2 and GluA4 in olfactory bulb astrocytes. The results indicate that olfactory bulb astrocytes possess both GluA2-containing and GluA2-lacking AMPA receptors, the latter being blocked by Naspm.

## Results

### Olfactory bulb astrocytes respond to AMPA application

**Figure 1:**
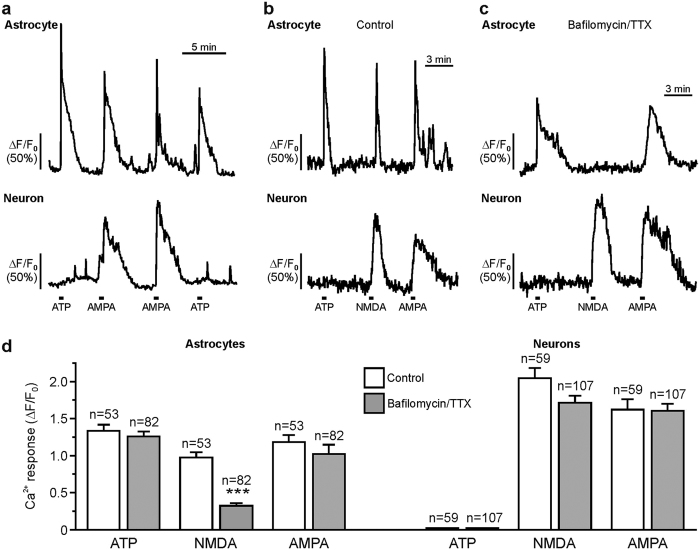
Bafilomycin A1 and TTX fail to reduce AMPA-induced Ca^2+^ transients in periglomerular astrocytes. (**a**) Ca^2+^ imaging traces of a single periglomerular astrocyte (upper trace) and neuron (lower trace) showing Ca^2+^ responses to ATP (100 μM) and AMPA (50 μM). (**b**) Ca^2+^ responses evoked by ATP (100 μM), NMDA (100 μM) and AMPA (50 μM) in the absence and (**c**) presence of TTX (1 μM) and bafilomycin A1 (10 μM). (**d**) Normalized and averaged amplitudes of Ca^2+^ transients evoked by ATP, NMDA and AMPA under control conditions (open bars) and in the presence of bafilomycin A1 and TTX (gray bars). ***p < 0.001.

We were interested whether olfactory bulb astrocytes respond to bath application of AMPA with Ca^2+^ signaling. We used application of ATP to test the viability of the cells and to identify astrocytes in the glomerular layer. Olfactory bulb astrocytes express P2Y_1_ receptors and respond to bath application of ATP and ADP with Ca^2+^ transients, whereas olfactory bulb neurons do not^21^. In the present study, 100 μM ATP evoked a Ca^2+^-dependent increase in Fluo-4 fluorescence by 1.34 ± 0.08 ΔF/F_0_ (n = 53) in periglomerular astrocytes, while in neurons, ATP did not evoke Ca^2+^ signaling (Fig. 1a). 59.3% of ATP-sensitive astrocytes and all neurons also responded to bath application of 50 μM AMPA (Fig. 1a). The mean Ca^2+^ increase was 1.18 ± 0.09 ΔF/F_0_ (n = 53) in AMPA-responding astrocytes and 1.62 ± 0.14 ΔF/F_0_ (n = 59) in neurons. To test whether AMPA evoked Ca^2+^ transients in periglomerular astrocytes directly or via activation of neurons and subsequent neurotransmitter release, we suppressed vesicular neurotransmitter release by incubation of the brain slices with 10 μM bafilomycin A1 (duration 40 min) and added 1 μM tetrodotoxin (TTX) to the artificial cerebrospinal fluid (ACSF) (Fig. 1b and c). We used NMDA as a control of successful TTX/bafilomycin treatment, since direct NMDA-induced stimulation of astrocytes is negligible and hence NMDA-evoked Ca^2+^ transients in astrocytes are expected to be suppressed by TTX/bafilomycin^22^. 100 μM NMDA evoked an increase in Ca^2+^ of 0.97 ± 0.07 ΔF/F_0_ (n = 53) under control conditions, while the increase was significantly reduced to 0.32 ± 0.03 ΔF/F_0_ (n = 82; p < 0.001) by treatment with TTX/bafilomycin (Fig. 1d). In contrast to NMDA, both ATP and AMPA induced Ca^2+^ transients in astrocytes in the presence of TTX/bafilomycin that did not differ in amplitude compared to transients evoked in the absence of TTX/bafilomycin (Fig. 1b and c). The amplitudes of the Ca^2+^ transients in the presence of TTX/bafilomycin were 1.26 ± 0.07 ΔF/F_0_ (n = 82; ATP; p = 0.479) and 1.02 ± 0.13 ΔF/F_0_ (n = 82; AMPA; p = 0.318). The results indicate that ATP and AMPA directly induced Ca^2+^ transients in astrocytes. Therefore, we used bafilomycin A1 and TTX in all following Ca^2+^ imaging experiments (except for electrical stimulation of axons) to isolate the direct response in astrocytes.

### Ca^2+^-permeable AMPA receptors mediate Ca^2+^ influx into periglomerular astrocytes

**Figure 2:**
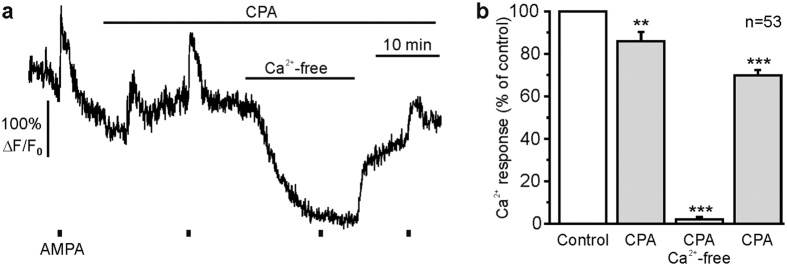
AMPA-induced changes in intracellular Ca^2+^ are not dependent on intracellular Ca^2+^ stores. (**a**) Ca^2+^ transient of a periglomerular astrocyte evoked by AMPA (25 μM) under control conditions, in the presence of CPA (20 μM), and in Ca^2+^-free saline in CPA. (**b**) CPA had only a small effect on AMPA-induced Ca^2+^ transients, while Ca^2+^ transients were entirely suppressed in Ca^2+^-free saline. **p < 0.01; ***p < 0.001.

AMPA can activate AMPA receptors as well as kainate receptors^25^, both of which may trigger Ca^2+^ influx through the receptor channel itself. In addition, AMPA/kainate receptors have been shown to stimulate G protein-coupled pathways, including Ca^2+^ release from intracellular stores^26^,^27^,^28^. To test whether Ca^2+^ is released from internal stores, we applied AMPA (25 μM) after Ca^2+^ stores were depleted by incubation with 20 μM cyclopiazonic acid (CPA). As shown in Fig. 2, the AMPA-induced Ca^2+^ response in periglomerular astrocytes was only slightly reduced after Ca^2+^ store depletion. In Ca^2+^-free, EGTA-buffered ACSF, however, the Ca^2+^ response was entirely blocked (n = 53; p < 0.001), indicating that AMPA induced Ca^2+^ influx from the extracellular space.

**Figure 3:**
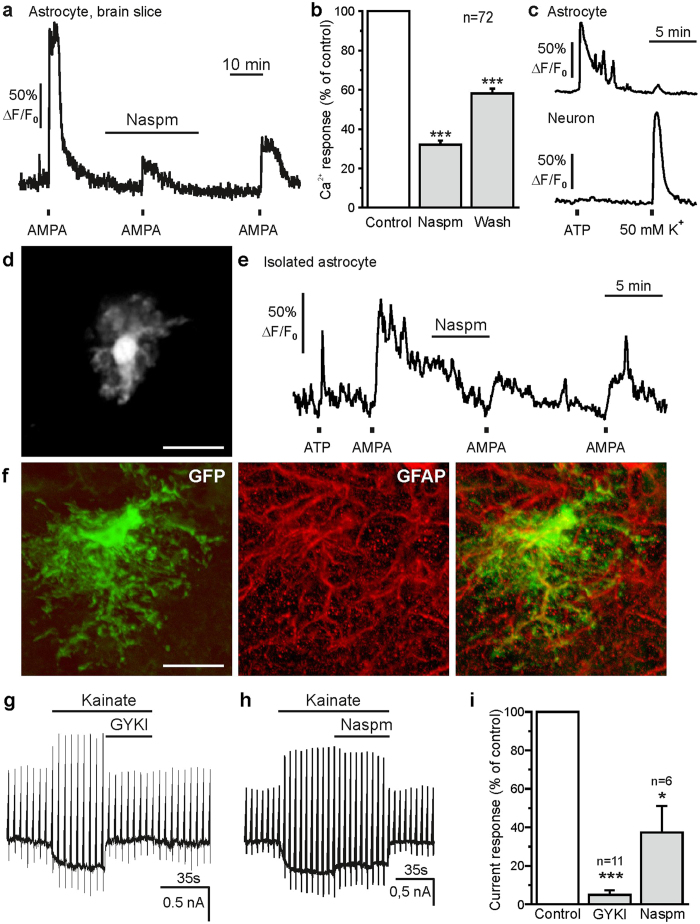
Naspm reduces AMPA receptor-induced Ca^2+^ transients and membrane currents in periglomerular astrocytes. (**a**) Effect of Naspm (50 μM) on AMPA-induced Ca^2+^ transients after incubation of brain slices in bafilomycin A1 and TTX. (**b**) Naspm significantly reduced AMPA-induced Ca^2+^ transients in periglomerular astrocytes. Wash out of Naspm led to a significant recovery of the Ca^2+^ response. (**c**) Increasing external K^+^ to 50 mM evoked large Ca^2+^ transients in neurons, but only small Ca^2+^ rises in astrocytes, indicating lack of voltage-gated Ca^2+^ influx in astrocytes. (**d**) Fluo-4-loaded isolated astrocyte. Scale bar: 20 μm. (**e**) Effect of Naspm (50 μM) on AMPA-evoked Ca^2+^ transients in an isolated olfactory bulb astrocyte. (**f**) Immunostaining of an eGFP-positive periglomerular astrocyte in an hGFAP-eGFP mouse (anti-GFP, green) and colabeling of GFAP as a marker for astrocytes (anti-GFAP, red). Scale bar: 10 μm. (**g**) Whole-cell current trace of a dissociated astrocyte recorded in BaCl_2_ (100 μM), quinine (100 μM) and cyclothiazide (100 μM). Kainate (500 μM) evoked an inward current that was entirely blocked by GYKI 53655 (100 μM), but (**h**) was only partly reduced by Naspm (50 μM). (**i**) Normalized averaged effects of GYKI 53655and Naspm on kainate-induced currents.

The AMPA-induced Ca^2+^ influx could be mediated by Ca^2+^-permeable AMPA receptors or by AMPA-evoked depolarization and subsequent activation of voltage-gated Ca^2+^ channels. To test whether AMPA evoked Ca^2+^ influx through Ca^2+^-permeable AMPA receptors we employed Naspm, a highly selective antagonist of GluA2-lacking, Ca^2+^-permeable AMPA receptors^29^,^30^. In the presence of 50 μM Naspm, AMPA-evoked Ca^2+^ transients were significantly reduced by 68.0 ± 2.2% (n = 72; p < 0.001) (Fig. 3a and b). In addition, depolarization of the cells by application of 50 mM K^+^ in the presence of bafilomycin A1 and TTX elicited only a small Ca^2+^ transient in astrocytes of 0.13 ± 0.09 ΔF/F_0_ (n = 16), leading to the conclusion that Ca^2+^ influx through voltage-gated Ca^2+^ channels is negligible (Fig. 3c). Hence, AMPA-evoked Ca^2+^ transients appear to be mainly mediated by Ca^2+^ influx through Ca^2+^-permeable AMPA receptors. We also used acutely isolated astrocytes to test the effect of Naspm on AMPA-evoked Ca^2+^ signaling. AMPA-evoked Ca^2+^ transients were significantly reduced by 50 μM Naspm (n = 31; p < 0.001), confirming the involvement of Ca^2+^-permeable AMPA receptors in Ca^2+^ signaling in olfactory bulb astrocytes (Fig. 3d and e). On average, 25 μM AMPA induced Ca^2+^ transients with an amplitude of 2.50 ± 0.50 ΔF/F_0_ (n = 31), which were reduced to 52.7 ± 5.3% by Naspm.

We aimed to verify these results in electrophysiological experiments on acutely dissociated astrocytes from the olfactory bulb glomerular layer, using hGFAP-eGFP reporter mice to identify astrocytes (Fig. 3f). We applied 500 μM kainate in the presence of 100 μM cyclothiazide to induce AMPA receptor-mediated membrane currents with minimal desensitization^12^,^31^. We and others have previously shown that activation of AMPA/KA receptors in glial cells leads to Na^+^ influx that plugs K^+^ channels^32^,^33^. To avoid that the kainate-induced receptor currents were obscured by a simultaneous block of Kir channels, BaCl_2_ was applied. Kainate application evoked an inward current of 427.8 ± 190.3 pA (n = 15) (at −70 mV) corresponding to a current density of 16.0 ± 5.1 pA/pF (n = 15) (Fig. 3f). 100 μM GYKI 53655 (1-(4-aminophenyl)-3-methylcarbamyl-4-methyl-3,4-dihydro-7,8-methylenedioxy-5H-2,3-benzodiazepine hydrochloride), a selective AMPA receptor blocker barely acting on kainate receptors^34^, entirely inhibited the kainate-induced inward current (n = 11; p < 0.001), confirming the activation of AMPA receptors by kainate in our experiments (Fig. 3g and i). In contrast to GYKI 53655, Naspm (50 μM) had variable effects on the kainate-induced inward current. In 2 out of 8 experiments, Naspm did not affect the kainate-evoked inward current, while in one experiment, Naspm entirely blocked the kainate-induced inward current. In the remaining 5 experiments, Naspm reduced the current by different amounts. In Naspm-sensitive cells, the kainate-induced inward current was significantly reduced by Naspm to 37.4 ± 13.7% of the control (n = 6; p = 0.031) (Fig. 3h and i). These results show that AMPA induced an influx of Ca^2+^ into periglomerular astrocytes that in the majority (75%) of cells is Naspm-sensitive and thus mediated by Ca^2+^-permeable AMPA receptors.

### Distribution of AMPA receptor subunits in the glomerular layer

**Figure 4:**
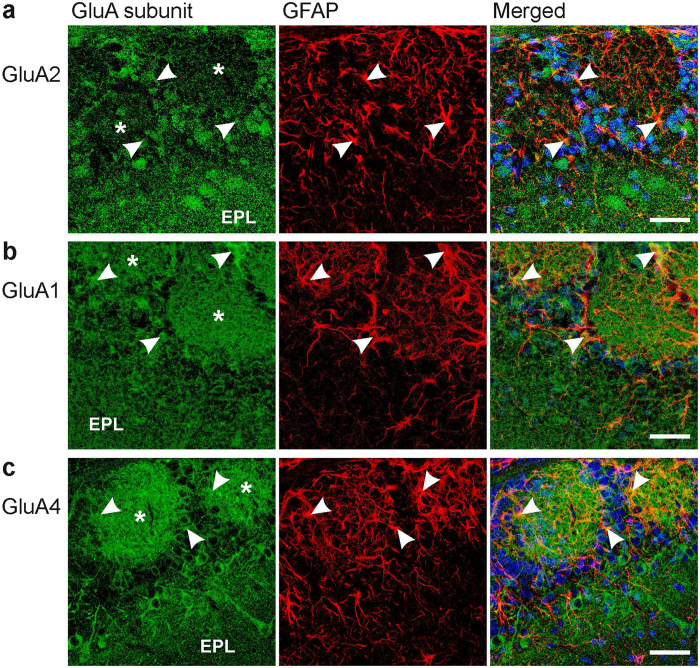
Immunostaining of AMPA receptor subunits in the olfactory bulb. (**a**) GluA2 immunoreactivity (green) was detected in the external plexiform layer (EPL) and in cell bodies surrounding glomeruli. Glomeruli are indicated by asterisks. Moderate GluA2 immunoreactivity was also found in astrocytes highlighted by GFAP immunoreactivity (red), as indicated by yellow pixels in the merged image. Arrows point to astrocyte structures that were colabeled with GluA immunoreactivity. Nuclei were stained with Hoechst 33342 (blue). (**b**) GluA1 and GFAP colocalization. (**c**) GluA4 and GFAP colocalization. Scale bars: 20 μm.

The Ca^2+^ permeability of AMPA receptors critically depends on the relative abundance of GluA2 within the channel complex^14^,^35^,^36^. Since our electrophysiological and Ca^2+^ imaging data suggest Ca^2+^-permeable AMPA receptors in periglomerular astrocytes, we investigated the cellular distribution of GluA2 in the olfactory bulb. GluA2 immunoreactivity was mainly found rather homogeneously in the external plexiform layer and in somata of periglomerular cells, whereas GluA2 immunoreactivity was much weaker in the synaptic neuropil in the core of the glomeruli (Fig. 4a). Only moderate colocalization with GFAP-positive periglomerular astrocytes was detected (Fig. 4a, merged image). We also investigated the distribution of GluA1 and GluA4. GluA3 was not investigated because it is barely expressed in the olfactory bulb^37^,^38^. GluA1 was widely distributed in the glomerular and the external plexiform layers, including the neuropil of glomeruli (Fig. 4b). Intense colocalization of GluA1 and GFAP was found in periglomerular astrocytes and their processes in the neuropil. GluA4 immunoreactivity resembled GluA1 immunoreactivity, with universal distribution in the external plexiform layer, the glomerular layer and the glomerular neuropil as well as clear colocalization with GFAP (Fig. 4c). We used cerebellar tissue of the same animals to verify specificity of antibody staining. The distribution of all three subunits investigated was in accordance with the distribution found in previous publications^39^,^40^,^41^,^42^; GluA1 was expressed in both neurons and glial cells, GluA2 exclusively in neurons and GluA4 predominantly in Bergmann glial cells (Supplementary Fig. S1).

### Endogenous glutamate release activates astrocytic Ca^2+^-permeable AMPA receptors

**Figure 5:**
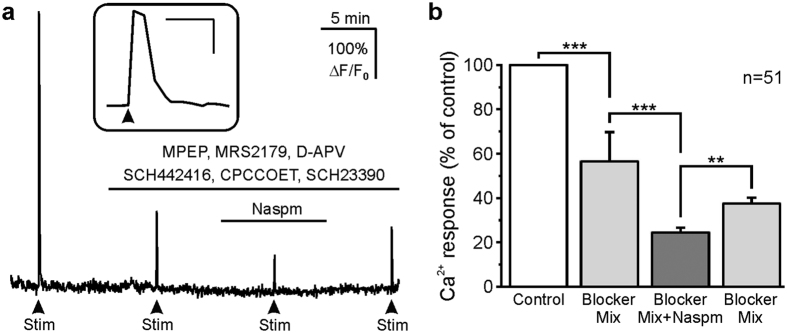
Electrical stimulation of axons of olfactory receptor neurons evokes Naspm-sensitive Ca^2+^ transients in GCaMP6s-expressing astrocytes. (**a**) Ca^2+^ transients evoked by electrical stimulation in the absence of receptor blockers, in the presence of a mix of receptor blockers (2 μM MPEP, antagonist of mGluR_5_; 30 μM MRS2179, P2Y_1_ receptor antagonist; 100 μM D-APV, NMDA receptor antagonist; 1 μM SCH442416, type 1 dopamine receptor antagonist; 100 μM CPCCOET, mGluR_1_ antagonist; 2 μM SCH23390, A_2A_ receptor antagonist) and after addition of Naspm (50 μM). Inset: First Ca^2+^ response (control) at larger time scale. Inset scale bars: 20 s, 200% ΔF/F_0_. (**b**) Normalized averaged amplitudes of Ca^2+^ rises evoked by electrical stimulation.

Olfactory receptor neurons (ORN) release glutamate from their axon terminals in the glomeruli, which evokes Ca^2+^ signaling in astrocytes^18^,^20^,^23^. We studied the effect of glutamate released upon electrical stimulation (10 Hz, 2 s) of ORN axons on astrocytic Ca^2+^ (Fig. 5). We crossbred Glast-CreERT2 and GCaMP6s^fl/fl^ mice to receive mice in which the genetic Ca^2+^ indicator GCaMP6s is specifically expressed by astrocytes. In brain slices of these mice, ORN stimulation resulted in Ca^2+^ transients with a mean amplitude of 3.51 ± 0.25 ΔF/F_0_ (n = 51) in periglomerular astrocytes. We aimed to isolate the putative Ca^2+^ response induced by activation of Ca^2+^-permeable AMPA receptors in astrocytes by applying a mix of receptor blockers antagonizing metabotropic neurotransmitter receptors known to induce Ca^2+^ transients in olfactory bulb astrocytes. In addition, we reduced glutamatergic activation of neurons and hence indirect effects with D-APV (D-2-amino-5-phosphonovaleric acid). This blocker mix significantly reduced the stimulation-induced Ca^2+^ response in periglomerular astrocytes to 56.4 ± 3.0% of the control (n = 51; p < 0.001) (Fig. 5a). Addition of Naspm (50 μM) further decreased the amplitude of stimulation-induced Ca^2+^ transients to 24.4 ± 2.2% of the control (n = 51). Ca^2+^ transients in the presence of Naspm were significantly smaller as compared to Ca^2+^ transients in the presence of the mix of blockers without Naspm (p < 0.001), indicating that glutamate release from OSN axons triggers Ca^2+^ signaling in periglomerular astrocytes via Ca^2+^-permeable AMPA receptors.

## Discussion

In the present study, we have investigated the role of AMPA receptors in Ca^2+^ signaling in periglomerular astrocytes of the olfactory bulb. Our results show that axonal stimulation activates AMPA receptor-mediated Ca^2+^ influx into periglomerular astrocytes. This Ca^2+^ influx was largely reduced by Naspm, a blocker of GluA2-lacking, Ca^2+^-permeable AMPA receptors^29^,^30^. Our antibody staining revealed expression of GluA1, GluA2 and GluA4 in periglomerular astrocytes, and patch-clamp recordings demonstrated only partial block of AMPA receptor-mediated inward currents by Naspm, suggesting that both GluA2-containing and GluA2-lacking AMPA receptors are expressed by periglomerular astrocytes.

Abundant expression of GluA1, GluA2 and GluA4 in the rodent olfactory bulb has been published before. GluA1, e.g., is mainly found in the external plexiform and the glomerular layers and is expressed by periglomerular neurons and mitral/tufted cells^39^,^43^,^44^,^45^. GluA2 has the highest expression of all GluA subunits in the olfactory bulb as assed by PCR^36^ and is located in mitral/tufted cells and granule cells^40^. GluA3 expression in the olfactory bulb is negligible, while GluA4 expression is moderate and GluA4 protein could be detected in the mitral cell layer, the external plexiform layer, the glomerular layer and the nerve layer^38^,^40^. However, all these studies focused on neurons, and none of them investigated the colocalization of the GluA subunits with astrocytes. We colabeled GluA immunostainings with an antibody against GFAP to highlight astrocytes in the glomerular layer. Colocalization of GluA subunits and GFAP was found for all subunits tested, with obvious colocalization for GluA1 and GluA4, but only moderate colocalization for GluA2. This suggests that only a fraction of AMPA receptors in periglomerular astrocytes comprise GluA2 subunits, while the remaining fraction of AMPA receptors lacks GluA2 subunits and hence is both Ca^2+^-permeable and Naspm-sensitive^35^,^36^,^46^. This is confirmed by the partial block of AMPA receptor currents by Naspm as measured in acutely dissociated, GFP-labeled astrocytes.

Several results indicate that in periglomerular astrocytes, AMPA directly gates Ca^2+^ influx. Firstly, AMPA-induced Ca^2+^ transients upon agonist bath application were not reduced by suppressing potential indirect effects (through neuronal transmitter release) with the sodium channel blocker TTX and bafilomycin, an inhibitor of vesicular H^+^ pumps required for filling synaptic vesicles with neurotransmitter molecules^47^. NMDA-evoked and high-K^+^-evoked Ca^2+^ transients in astrocytes, in contrast, were greatly reduced by TTX/bafilomycin, in line with the notion that olfactory astrocytes do not significantly express voltage-gated Ca^2+^ channels and NMDA receptors and hence high-K^+^-evoked and NMDA-evoked Ca^2+^ signaling was mainly due to neuronal transmitter release. Whether the remaining, TTX/bafilomycin-insensitive part of NMDA-evoked Ca^2+^ transients reflects insufficient efficacy of TTX/bafilomycin or expression of NMDA receptors in olfactory bulb astrocytes, as demonstrated for cortical astrocytes and for oligodendrocytes, remains to be shown^48^,^49^,^50^,^51^,^52^,^53^. In addition, TTX/bafilomycin-insensitive neurotransmitter release such as reversal of neurotransmitter uptake due to the NMDA-evoked increase in Na^+^ in neurons and subsequent activation of astrocytic receptors might also contribute to NMDA-evoked Ca^2+^-transients in astrocytes. Such TTX/bafilomycin-insensitive neurotransmitter release could contribute to astrocytic Ca^2+^ signalling not only upon application of NMDA, but also AMPA. However, it is very unlikely that this has a major contribution to the AMPA-evoked Ca^2+^ signaling, since the same mechanism is activated during NMDA and high K^+^ application, which produced only small Ca^2+^ transients in the presence of TTX/bafilomycin.

Secondly, AMPA receptor activation did only weakly evoke Ca^2+^ release from internal stores, but induced Ca^2+^ transients that mainly depended on the presence of Ca^2+^ in the bath solution, indicating Ca^2+^ influx. Besides Ca^2+^ influx through AMPA receptors, an increase in Ca^2+^ due to inhibition or reversal of Na^+^/Ca^2+^ exchanger (NCX) upon the AMPA-evoked Na^+^ increase in astrocytes might contribute to the Ca^2+^ transients triggered by AMPA application, as shown before for Na^+^ increases evoked by GABA transport into olfactory bulb and hippocampal astrocytes^22^,^54^. In olfactory bulb astrocytes, NCX-dependent Ca^2+^ increases were small yet sufficient to trigger Ca^2+^-induced Ca^2+^ release, which was abolished upon store depletion with CPA^22^. AMPA-evoked Ca^2+^ transients in the present study, in contrast, were only weakly affected by store depletion, indicating that they were not mainly mediated by NCX-dependent Ca^2+^-induced Ca^2+^ release.

Thirdly, our findings were confirmed with acutely dissociated cells excluding indirect effects through neuronal activation. The observation that AMPA receptor currents were sensitive to Naspm further substantiated a Ca^2+^ permeability of the receptors. It should be noted that during our patch-clamp experiments, AMPA receptor-evoked currents were isolated by blocking potassium conductances with Ba^2+^ and quinine, which affect membrane properties of astrocytes^55^,^56^ and suppress Na^+^-dependent block of K^+^ channels^32^,^33^; hence, in the absence of K^+^ channel blockers, AMPA receptor-evoked effects on membrane currents might be more complex than shown in our experiments. Importantly, we have demonstrated that Ca^2+^-permeable AMPA receptors of periglomerular astrocytes are activated through electrical stimulation of ORN axons. ORN axons release glutamate and ATP^18^,^19^,^21^,^57^, and electrical stimulation of ORN axons as well as odor stimulation of ORN has been shown to trigger Ca^2+^ signaling in periglomerular astrocytes by activation of mGluR_5_, P2Y_1_ and A_2A_ receptors^20^,^21^. However, in the present study we have inhibited these receptors as well as dopamine receptors, which in the olfactory bulb are expressed by many neurons^58^,^59^,^60^, to reduce potential indirect effects via dopaminergic neurons. The stimulation-induced Ca^2+^ response in periglomerular astrocytes remaining in this blocker cocktail was sensitive to Naspm, indicating that this form of axon-glia interaction activates Ca^2+^-permeable AMPA receptors. Olfactory bulb astrocytes have been shown to mediate neurovascular coupling^20^,^22^,^23^ as well as release of glutamate, GABA and ATP affecting mitral cells and granule cells^24^,^61^. Accordingly, astrocytes might sense the level of axonal activity by gradual activation of Ca^2+^ influx through their AMPA receptors, thereby modulating gliotransmitter release and adapting local circulation and energy supply to the actual metabolic requirements. In addition, astrocytes are involved in the development of the glomeruli, and AMPA receptor-mediated astrocyte Ca^2+^ signaling might affect neurite growth and synaptogenesis^62^,^63^.

## Material and Methods

### Animals used for slice preparation

For Ca^2+^ imaging experiments, NMRI, GCaMP6s^fl/fl^ and GLAST-CreETR2 mice of both genders at postnatal days 14 to 40 were used^64^,^65^. NMRI and GLAST-CreETR2xGCaMP6s^fl/fl^ mice were raised in the animal facility at the University of Hamburg (Germany). hGFAP-eGFP mice^66^ used for electrophysiology and immunofluorescence staining were obtained from the animal facility at the University of Bonn Medical Center (Germany). All experiments were performed in accordance with EU and local animal welfare guidelines and were approved by the state’s animal welfare committee (GZ G21305/591-00.33; Behörde für Gesundheit und Verbraucherschutz, Hamburg, Germany). Olfactory bulbs were prepared and sliced (VT1200, Leica, Benzheim, Germany) in cooled, carbogen-gassed preparation solution and transferred to carbogen-gassed ACSF at 30 °C for recovery.

### Solutions and chemicals

The following solutions were employed (molarity in mM), ACSF: 120 NaCl, 2.5 KCl, 1 NaH_2_PO_4_ × 2H_2_O, 26 NaHCO_3_, 2.8 D-(+)-glucose, 1 MgCl_2_, 2 CaCl_2_; Ca^2+^-free ACSF: 120 NaCl, 2.5 KCl, 1 NaH_2_PO_4_ × 2H_2_O, 26 NaHCO_3_, 2.8 D-(+)-glucose, 3 MgCl_2_, 0.5 EGTA; preparation solution for Ca^2+^ imaging: 83 NaCl, 1 NaH_2_PO_4_ × 2H_2_O, 26.2 NaHCO_3_, 2.5 KCl, 70 Sucrose, 20 D-(+)-glucose, 2.5 MgSO_4_ × 7 H_2_O; preparation solution for electrophysiology: 87 NaCl, 1.25 NaH_2_PO_4_ × 2H_2_O, 25 NaHCO_3_, 2.5 KCl, 7 MgCl_2_ × 6 H_2_O, 0.5 CaCl_2_ × 6 H_2_O, 60 Sucrose, 25 D-(+)-glucose (325 mOsm). ACSF, preparation solutions and Ca^2+^-free ACSF were continuously gassed with carbogen (95% O_2_, 5% CO_2_) to buffer the pH at 7.4 and to supply oxygen. Patch clamp-recording of isolated cells was performed in bath solution containing (in mM): 150 NaCl, 5 KCl, 2 MgCl_2_ × 6 H_2_O, 2 CaCl_2_ × 6 H_2_O, 10 D-(+)-glucose, 10 HEPES, pH 7.4, gassed with oxygen. The compounds amino-3-hydroxy-5-methyl-4-isoxazolephosphonic acid (AMPA), cyclothiazide, (E)-ethyl 1,1a,7,7a-tetrahydro-7-(hydroxyimino)cyclopropa[b]chromene-1a-carboxylate (CPCCOEt), 2-methyl-6-(phenylethynyl)pyridine (MPEP), 2′-deoxy-N6-methyladenosine 3′,5′-bisphosphate (MRS2179), 2-(2-furanyl)-7-[3-(4-methoxyphenyl)propyl]-7H-pyrazolo[4,3-e][1,2,4]triazolo[1,5-c]pyrimidin-5-amine (SCH442416) and (R)-(+)-7-chloro-8-hydroxy-3-methyl-1-phenyl-2,3,4,5-tetrahydro-1H-3-benzazepine (SCH23390) were obtained from Abcam (Cambridge, United Kingdom). Adenosine 5′-triphosphate (ATP), kainate, N-methyl-D-aspartic acid (NMDA) and papain was purchased from Sigma Aldrich (Taufkirchen, Germany), GYKI 53655 from Tocris (Bristol, UK). Bafilomycin A1 and CPA were acquired from Enzo Life Sciences (Lörrach, Germany). The reagents D-APV, Naspm and TTX were received from Alomone labs (Jerusalem, Israel). All reagents were stored as stock solutions corresponding to the manufacturer’s instructions and added to ACSF directly before the experiment.

### Ca^2+^ imaging

Tissue slices were placed in a recording chamber and fixed with a platinum frame fitted with nylon strings. Slices were incubated with the membrane-permeable form of the Ca^2+^ indicator Fluo-4 (Fluo-4-AM; 2 μM in ACSF) made from a 4-mM stock solution (dissolved in DMSO and 20% pluronic acid) for 40 min. In most of the experiments, bafilomycin A1 (10 μM) was added to the Fluo-4-AM solution. In some experiments, GCaMP6s fluorescence was used as an indicator of Ca^2+^ concentration. Changes in intracellular Ca^2+^ levels in periglomerular astrocytes were recorded by confocal microscopy (C1 Eclipse, Nikon, Düsseldorf, Germany). An excitation laser wavelength of 488 nm and a frame rate of 0.3–0.5 fps were used. Drugs were administered via the perfusion system except for AMPA. The application solution containing AMPA was applied directly into the perfusion stream in the bath with a custom made application system to allow for a semi-fast application (within 3–5 s). The flow rate of the application system equaled the flow rate of the perfusion system, resulting in a 1:2 dilution of the agonist concentration as adjusted in the application solution.

For electrical stimulation of axons in tissue slices, a glass pipette with a resistance of 2–2.5 MΩ filled with ACSF was used. The pipette was placed on the surface of the olfactory nerve layer (ONL) comprising axons of olfactory receptor neurons and 250-μA stimuli were applied for 2 s at 10 Hz.

### Isolation of periglomerular astrocytes

For tissue preparation, 300 μm thick sagittal slices from the olfactory bulb were prepared from hGFAP-eGFP mice (postnatal day 8–14) in ice cold, carbogen (95% O_2_/5% CO_2_) gassed ACSF supplemented with sucrose (preparation solution) using a vibratome (VT1200S, Leica, Nussloch, Germany). Slices were transferred for 15 min to warm (35 °C) preparation solution and then to standard ACSF (room temperature, 1 h). Cells were isolated using an enzymatic/mechanical approach^13^. Slices were incubated in ACSF supplemented with papain (1.5 mg/ml; 24 U/ml) (Sigma, Taufkirchen, Germany) and L-cysteine (0.35 mg/ml) (Sigma) (10 min) and continuously bubbled with carbogen. Subsequently, slices were transferred to the recording solution and the glomerular layer was dissected under a stereomicroscope (KL200, Zeiss, Jena, Germany). Cells were isolated in the recording chamber with tungsten needles and Pasteur pipettes, and allowed to settle for 15 min before analysis. Periglomerular astrocytes were identified by their green fluorescence and morphology. For Ca^2+^ imaging experiments, astrocytes were isolated from NMRI mice, seeded on concanavalin A-coated cover slipes, loaded with Fluo-4-AM (2 μM in ACSF) for 30–45 min and imaged as discribed for brain slices.

### Electrophysiological recordings

Experiments on isolated cells employed a customized concentration clamp device connected to an EPC-7 amplifier and TIDA software (Heka Lambrecht, Germany) as described elsewhere^13^. Astrocytes were visualized with an inverted microscope (Axiovert 135, Zeiss) equipped with DIC and epifluorescence. Pipettes were manufactured from borosilicate glass (2–4 MΩ; Science Products, Hofheim, Germany) and filled with a solution containing (in mM): 130 KCl, 0.5 CaCl_2_, 2 MgCl_2_, 5 BAPTA, 10 HEPES, and 3 Na_2_-ATP, 0.05 spermine, pH 7.25. Currents were sampled at 0.1 to 30 kHz and filtered at 3 or 10 kHz. Holding potential was −70 mV. Input and series resistance were continuously checked by applying 10 mV test pulses. The liquid junction potential was not corrected for. Recordings were performed at room temperature. To separate the AMPA receptor conductance from simultaneously occurring changes in K^+^ conductance, drug application to isolated cells was performed in HEPES-buffered recording solution, supplemented with the K^+^ channel blockers quinine (100 μM) and BaCl_2_ (100 μM)^33^.

### Data analysis

To analyze changes of the Ca^2+^ level in astrocytes, cell somata were marked as regions of interests (ROIs) using EZC1 Viewer software (Nikon). Cells located in the glomerular layer that showed a Ca^2+^ response to ATP were identified as periglomerular astrocytes^21^. To analyze changes of Ca^2+^ levels over time, Fluo-4 and GCaMP6s fluorescence intensity (F), respectively, was recorded throughout the experiment and normalized to the basal fluorescence intensity in absence of stimuli (F_0_). Changes in Ca^2+^ are given by ΔF/F_0_. All values are given as mean values ± standard error of the mean. Data for every set of experiment was acquired from at least three different animals. The assessment of statistical significance by comparing two means was done by Student’s t-test or, if applicable, one-way ANOVA with Fisher’s post-hoc test at an error probability p (*p < 0.05; **p < 0.01; ***p < 0.001).

### Immunohistology

Immunohistological staining was performed on 100-μm thick sagittal slices of olfactory bulbs of NMRI and hGFAP-eGFP mice. For anti-GluA staining, cerebella of the same animals were used as a positive control to verify specific antibody staining. After dissection, olfactory bulbs and cerebella were stored and refrigerated in paraformaldehyde solution (PFA, 4%) in phosphate buffered solution (PBS) containing (in mM): 130 NaCl, 7 Na_2_HPO_4_, 3 NaH_2_PO_4_. Slices were cut with a vibratome (VT1000, Leica) and incubated with the primary antibodies anti-GluA1 (guinea pig; 1:200; Alomone Labs), anti-GluA2 (rabbit; 1:200; Millipore, Darmstadt, Germany), anti-GluA4 (rabbit; 1:200; Millipore), anti-GFAP (rabbit, 1:000, Dako, Hamburg, Germany), anti-GFAP (chicken; 1:500; Abcam) and anti-GFP (chicken, 1:500, SySy, Göttingen, Germany). Antibodies were diluted in 1% NGS, 0.05% TritonX100 in PBS. Subsequently, slices were incubated with secondary antibodies over night at room temperature. Secondary antibodies (1:1000 in PBS) used were: goat anti-rabbit Alexa Fluor 488 (Invitrogen Thermo Fisher, Darmstadt, Germany), goat anti-rabbit Alexa Fluor 555 (Invitrogen Thermo Fisher), goat anti-chicken Alexa Fluor 555 and goat anti-chicken Alexa 488 (Abcam), CF488A donkey anti-guinea pig (Sigma-Aldrich). Hoechst 33342 (5 μM; Molecular Probes, Eugine, USA) was used for nuclear staining. Stacks of confocal images were acquired (C1 Eclipse, Nikon) using a 40x/NA 1.3 oil immersion lens. The axial step size was 150 nm. Image stacks of GluA staining were deconvolved using Huygen’s software (SVI, Hilversum, Netherlands). Projections were made using Image J (NIH, Bethesda, USA) and adjusted for brightness and contrast.

## Additional Information

**How to cite this article:** Droste, D. *et al*. Ca^2+^-permeable AMPA receptors in mouse olfactory bulb astrocytes. *Sci. Rep.* **7**, 44817; doi: 10.1038/srep44817 (2017).

**Publisher's note:** Springer Nature remains neutral with regard to jurisdictional claims in published maps and institutional affiliations.

## Supplementary Material

Supplementary Figure
