# Supplementary material for: Ca2+-permeable AMPA receptors in mouse olfactory bulb astrocytes
Source: Sci Rep. 2017 Mar 21;7:44817. doi: 10.1038/srep44817 (PMC5359673; doi:10.1038/srep44817)
Supplement: Supplementary Figure [file srep44817-s1.pdf]

Ca^{2+}-permeable AMPA receptors in mouse olfactory bulb astrocytes

Damian Droste, Gerald Seifert, Laura Seddar, Oliver Jädtke, Christian Steinhäuser, Christian Lohr

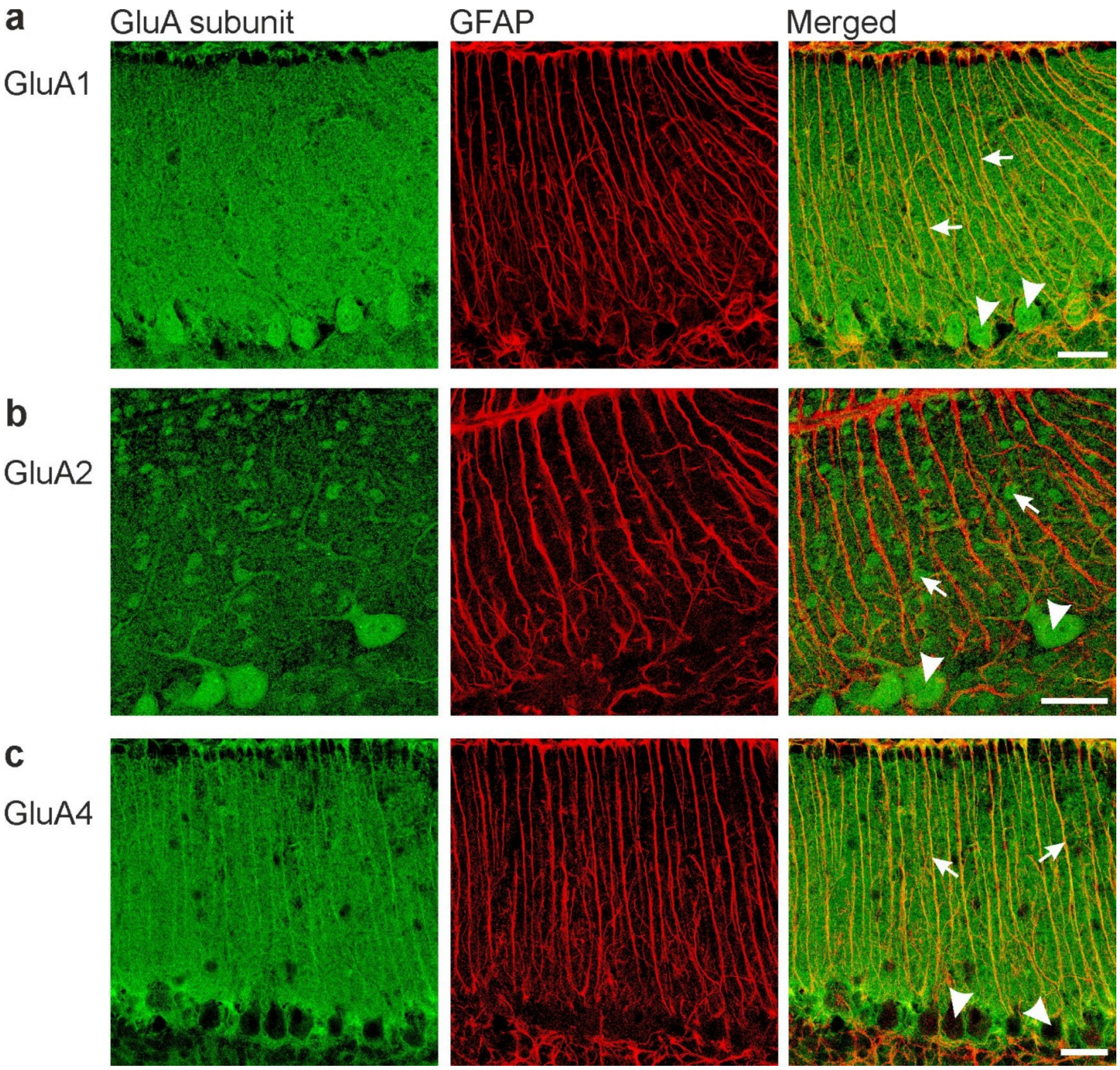

Supplementary Fig. S1. Antibody staining of cerebellar tissue. **a**) GluA1 antibodies (green) labeled Purkinje cells (arrowheads) and Bergmann glial cells (arrows). **b**) GluA2 immunoreactivity was detected in Purkinje cells (arrowheads) and interneurons (arrows). **c**) GluA4 immunoreactivity was detected in Bergmann glial cells (arrows), whereas Purkinje cells were not labeled (arrowheads). Scale bars: 20 μm.
